# Sphingosine-1-Phosphate Enhances α_1_-Adrenergic Vasoconstriction via S1P2–G_12/13_–ROCK Mediated Signaling

**DOI:** 10.3390/ijms20246361

**Published:** 2019-12-17

**Authors:** Cecília R. Panta, Éva Ruisanchez, Dorottya Móré, Péter T. Dancs, Andrea Balogh, Ágnes Fülöp, Margit Kerék, Richard L. Proia, Stefan Offermanns, Gábor J. Tigyi, Zoltán Benyó

**Affiliations:** 1 Institute of Translational Medicine, Semmelweis University, 1094 Budapest, Hungarymoredoro@gmail.com (D.M.); dancs.peter@med.semmelweis-univ.hu (P.T.D.); balogh.andrea@med.semmelweis-univ.hu (A.B.); nagy.margit@med.semmelweis-univ.hu (M.K.); gtigyi@uthsc.edu (G.J.T.); 2 National Institute of Diabetes and Digestive and Kidney Diseases (NIDDK), Bethesda, MD 20892, USA; richard.proia@nih.gov; 3 Max Planck Institute for Heart and Lung Research, 61231 Bad Nauheim, Germany; stefan.offermanns@mpi-bn.mpg.de; 4 Department of Physiology, University of Tennessee Health Science Center, Memphis, TN 38163, USA

**Keywords:** sphingosine-1-phosphate, α_1_-adrenoreceptors, vascular smooth muscle, hyperreactivity, S1P2 receptors, G _12/13_ proteins, Rho kinase, cardiovascular disease

## Abstract

Sphingosine-1-phosphate (S1P) has been implicated recently in the physiology and pathology of the cardiovascular system including regulation of vascular tone. Pilot experiments showed that the vasoconstrictor effect of S1P was enhanced markedly in the presence of phenylephrine (PE). Based on this observation, we hypothesized that S1P might modulate α_1_-adrenergic vasoactivity. In murine aortas, a 20-minute exposure to S1P but not to its vehicle increased the E_max_ and decreased the EC_50_ of PE-induced contractions indicating a hyperreactivity to α_1_-adrenergic stimulation. The potentiating effect of S1P disappeared in S1P2 but not in S1P3 receptor-deficient vessels. In addition, smooth muscle specific conditional deletion of G_12/13_ proteins or pharmacological inhibition of the Rho-associated protein kinase (ROCK) by Y-27632 or fasudil abolished the effect of S1P on α_1_-adrenergic vasoconstriction. Unexpectedly, PE-induced contractions remained enhanced markedly as late as three hours after S1P-exposure in wild-type (WT) and S1P3 KO but not in S1P2 KO vessels. In conclusion, the S1P–S1P2–G_12/13_–ROCK signaling pathway appears to have a major influence on α_1_-adrenergic vasoactivity. This cooperativity might lead to sustained vasoconstriction when increased sympathetic tone is accompanied by increased S1P production as it occurs during acute coronary syndrome and stroke.

## 1. Introduction

In spite of the relatively brief history of sphingosine-1-phosphate (S1P) as a biologically active lipid mediator, numerous reports highlight its major regulatory function in embryogenesis [1,2], the immune system and inflammation [3,4,5], the cardiovascular system [6,7,8,9,10], the nervous system [11,12,13], oncogenesis [14,15,16,17], as well as cellular motility and migration [18,19,20,21]. In the cardiovascular system, S1P has been implicated in the regulation of vasculo- and angiogenesis [22,23] and endothelial barrier function [24,25,26]. The role of S1P in regulating vascular tone remains controversial because studies conducted in different vascular regions of various animal species have often produced contradictory results [10]. Some publications reported a vasodilator effect of S1P [27,28,29,30,31], whereas others observed vasoconstriction and increased vascular tone as a result of S1P administration in different vascular regions [32,33,34,35,36,37]. In the present study, we hypothesized that the variability of S1P effects might be due in part to the absence or presence of other vasoactive mediators.

Data from previous studies indicate that the vascular effect of S1P is highly dependent on other concomitant factors and conditions. For example, the integrity or damage of the endothelium, the presence of different cells adhering to the endothelium, and the substances these adherent cells release might significantly modulate the S1P effect. In addition, specific characteristics of regional blood flow regulation can also modify the effect of S1P on vascular tone, leading to different effects of S1P in the pulmonary, uterine, splanchnic, and cerebral circulation systems [9,31,38,39,40,41]. Highly variable expression S1P receptors, and the coupling of S1P receptors to multiple intracellular signaling pathways, are further complicating factors [6,42]. These factors might also be responsible for activating the same receptor subtype, resulting in multiple divergent effects of S1P in different organs and vascular regions [7,9].

It is generally accepted that, of the five S1P receptors subtypes, S1P1, S1P2 and S1P3 are expressed most abundantly in the cardiovascular system. Activation of S1P1 and S1P3 has been shown to induce endothelium-dependent vasodilation [9,32]. However, in cerebral arteries, vasoconstriction has been linked to S1P3 activation [41]. Some publications on the vascular effects of S1P reported the results of in vitro experiments, whereas others described the results of in vivo studies. Results from in vivo studies tend to show higher variability, supporting the notion that, under these conditions the effect of S1P is influenced by a multitude of different co-existing factors.

In pilot experiments, we found that the vasoconstrictor effect of S1P was markedly enhanced with concomitant α_1_-adrenoreceptor activation. Based on these observations, we hypothesized that S1P might sensitize vessels to α_1_-adrenergic stimulation, and consequently, vasoactivity of S1P might be highly dependent on the sympathetic tone under in vivo conditions. Our results support this hypothesis by demonstrating an interaction between S1P with α_1_-adrenoreceptor agonists and also demonstrate the role of the S1P2–G_12/13_ –Rho-associated protein kinase (ROCK) signaling pathway in mediating the effect of S1P.

## 2. Results

**Figure 1:**
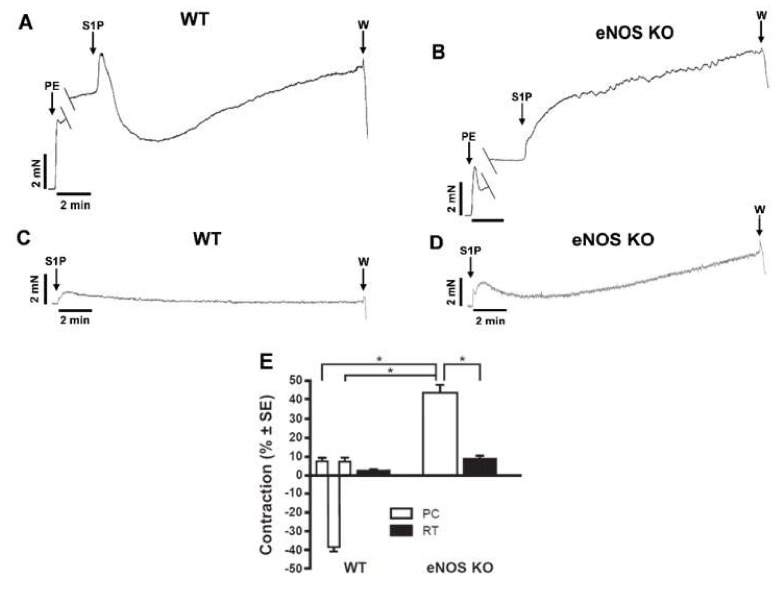
Effects of S1P on the vascular tone (**A–D**). Representative recordings of thoracic aorta (TA) segments prepared from WT and eNOS KO mice. “PE” and “S1P” marks administration of phenylephrine (PE) and sphingosine-1-phosphate (S1P), respectively. “W” denotes wash-out with fresh Krebs solution. In precontracted WT segments, S1P evoked an immediate, transient vasoconstriction, then a marked vasorelaxation, followed by sustained vasoconstriction (**A**). In contrast, S1P elicited a very strong vasoconstriction without vasorelaxation in eNOS KO vessels (**B**). S1P applied on the resting tone (RT) without PE-induced precontraction resulted in only minor changes of vascular tone (**C**,**D**). Statistical analysis of the vasoactive effects of S1P applied on the resting tone (RT) or after precontraction (PC) (**E**). * *p* < 0.01; 2-way ANOVA with Tukey’s *post hoc* test (*n* = 21–26).

First, we aimed to characterize the vasoactive effects of S1P in our experimental model. In phenylephrine (PE) precontracted mouse aortic segments, S1P usually evoked a tri-phasic response consisting of a transient constriction followed by a marked relaxation and finally a tonic constriction (Figure 1A). In order to dissect the relaxant and constrictor components of the response, we tested the effects of S1P in vessels of endothelial nitric oxide synthase knockout (eNOS KO) animals, as endothelial NO has been reported to mediate S1P-induced vasorelaxation [9,32]. In the absence of eNOS, S1P evoked strong and sustained vasoconstriction (Figure 1B). In order to gain in depth insight into the vasoconstrictor effect, these experiments were repeated with administration of S1P on the resting tone (RT) of the vessels. Interestingly, S1P induced only minor vasoconstriction in both wild-type (WT) (Figure 1C) and eNOS KO (Figure 1D) vessels. Taken together, these observations indicated that S1P is a weak vasoconstrictor by itself. However, it can significantly enhance the contractile effect of α_1_-adrenoreceptor stimulation (Figure 1E).

**Figure 2:**
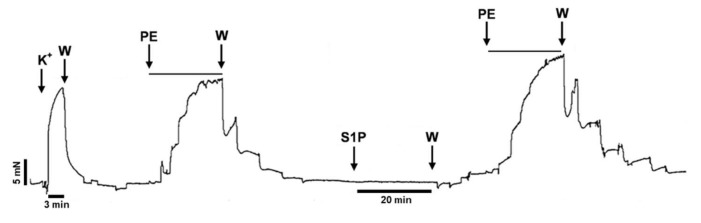
Original recording demonstrating the potentiating effect of S1P on PE-induced vasoconstriction in TA segments prepared from WT mice. “PE”, “S1P”, and “K^+^”denotes administration of the corresponding compounds and 124 mM potassium, respectively. “W” denotes wash-out with fresh Krebs solution. PE was administered at increasing concentrations (0.1 nM–10 µM) enabling the evaluation of the dose-response-relationship. Between two PE administrations, S1P (5 µM) was applied for 20 min followed by W.

To further test this hypothesis, we designed experiments to evaluate α_1_-adrenoreceptor-mediated vasoconstriction before and after incubation of the vessels with S1P. Indeed, PE-induced vasoconstriction of the vessels increased markedly after exposure to S1P (Figure 2).

**Figure 3:**
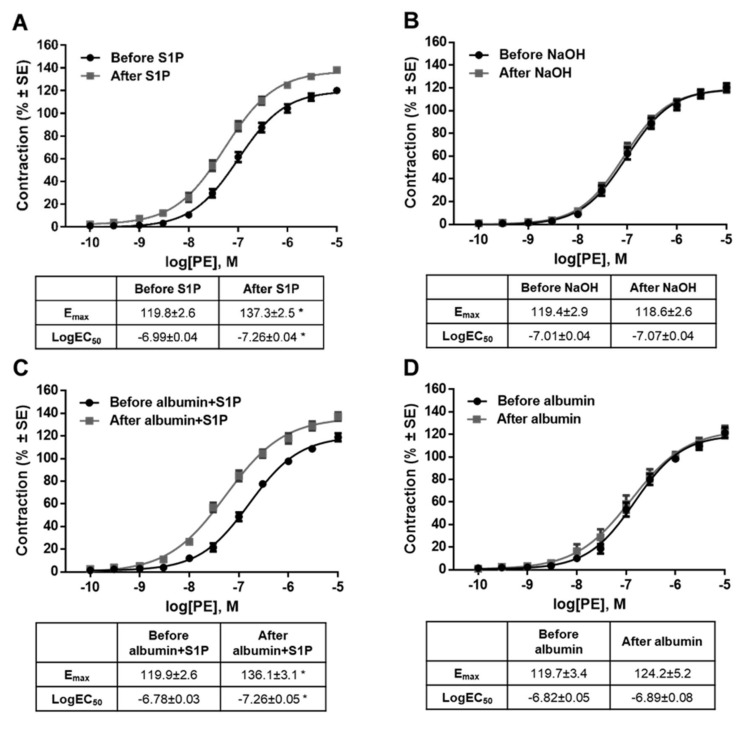
Effects of S1P (**A**,**C**) or its vehicle, (0.3 N NaOH or 5% albumin) (**B**,**D**) on α_1_-adrenoceptor-mediated vasoconstriction. Administration of S1P increased the contraction responses to PE. As a result, the dose-response curve shifted to the left and upwards resulting in increased E_max_ and decreased logEC_50._ This indicates that both the potency and efficacy were increased after incubation with S1P. The potentiating effect did not appear after incubation with vehicle. * *p* < 0.05 vs. before S1P (*n* = 5–41).

It is important to note that the potentiating effect of S1P on PE-induced contraction was observed after washing S1P out of the organ chamber. Statistical analysis revealed that the E_max_ value of the PE effect increased and the EC_50_ decreased significantly after S1P (Figure 3A), whereas the vehicle of S1P failed to induce any changes (Figure 3B). Because the S1P effects were reported to be highly vehicle-dependent in a recent study [43], we repeated our experiments using albumin as a carrier of S1P. Consistent with previous findings, S1P increased both the potency and efficiency of PE-induced vasoconstriction (Figure 3C), whereas its vehicle produced no effect (Figure 3D).

**Figure 4:**
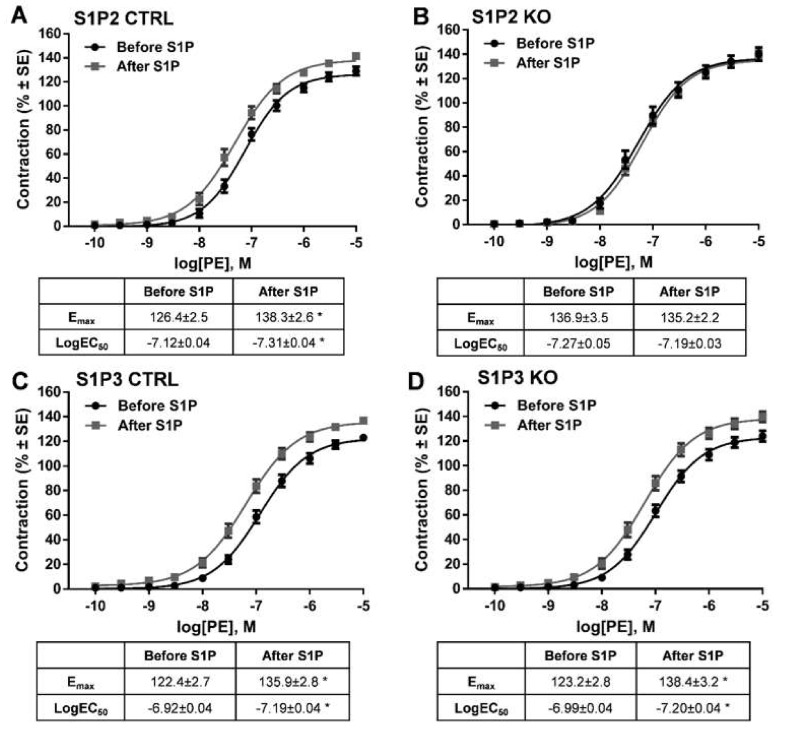
Identification of the receptor that mediates the potentiating effects of S1P. Following administration of S1P, the α_1_-adrenoceptor-mediated vasoconstriction increased markedly in S1P2 CTRL (**A**) but not in S1P2 KO vessels (**B**). In contrast, deletion of S1P3 failed to influence the effect of S1P (**C**,**D**). * *p* < 0.05 vs. before S1P (*n* = 8–25).

Our next aim was to identify the receptor subtype mediating S1P-induced potentiation of α_1_-adrenergic vasoconstriction. Previous studies showed that both S1P2 and S1P3 receptors can mediate the effects of S1P on vascular smooth muscle cells [9,32]. Therefore, we tested vessels isolated from S1P2 KO and S1P3 KO animals and their corresponding controls. Control vessels showed marked potentiation of PE-induced vasoconstriction after incubation with S1P (Figure 4A,C) resembling our previous observations in WT vessels (Figure 3A). In contrast, the potentiating effect of S1P failed to develop in S1P2 KO (Figure 4B), whereas it remained unaltered in S1P3 KO vessels (Figure 4D). These observations unambiguously indicate the exclusive role of S1P2 in mediating the enhanced response to PE. Interestingly, the contractile effect of PE already appeared to be higher before S1P administration in S1P2 KO vessels as compared with the controls. In order to test whether this hyperreactivity could by itself prevent further potentiation of the contractile response by S1P, we evaluated the effects of the thromboxane prostanoid receptor agonist U46619 on α_1_-adrenergic vasoconstriction. As U46619 was able to potentiate the effects of PE in S1P2 KO vessels (Figure 4E). We can conclude that these vessels did not lose their ability to develop hyperreactivity upon certain stimulation (Figure S1).

**Figure 5:**
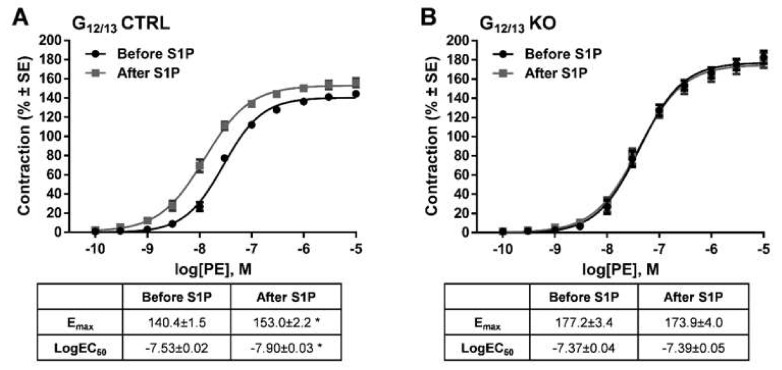
Identification of the G-protein involved in the signal transduction pathway mediating the potentiating effect of S1P. In G_12/13_ control (G_12/13_ CTRL vessels), the potentiating effect of S1P was clearly detectable (**A**), whereas the lack of G_12/13_ proteins (G_12/13_ KO) completely abolished it (**B**). * *p* < 0.05 vs. before S1P (*n* = 13–22).

In order to identify the intracellular signaling pathway mediating the effects of S1P, vessels deficient for Gα_12_ and Gα_13_ proteins in smooth muscle cells were examined. Whereas control vessels showed the potentiating effect of S1P on α_1_-adrenergic vasoconstriction (Figure 5A), this effect was abolished completely in vascular segments of G_12/13_ KO mice (Figure 5B), indicating the major role of G_12/13_ signaling in S1P-induced vascular hyperreactivity.

**Figure 6:**
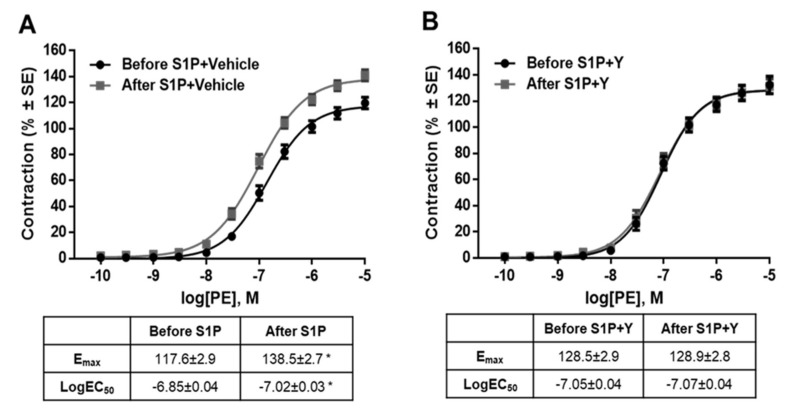
Downstream signaling of the potentiating effect of S1P. Co-administration of the ROCK inhibitor Y-27632 (2 µM) eliminated the potentiating effect of S1P (**B**), whereas its vehicle failed to influence the effect of S1P (**A**). * *p* < 0.05 vs. before S1P (*n* = 4–15).

As G_12/13_ proteins are often linked to the Rho–Rho-kinase (ROCK) signaling pathway, we hypothesized that ROCK was involved in S1P-induced modulated vascular reactivity. Indeed, the ROCK inhibitor Y-27632 prevented the development of S1P-induced potentiation (Figure 6B), whereas its vehicle showed no effect (Figure 6A). In addition, the structurally-unrelated ROCK inhibitor fasudil was also able to prevent S1P-induced hyperreactivity (Figure S2), proving the major role of ROCK in mediating the effect of S1P.

**Figure 7:**
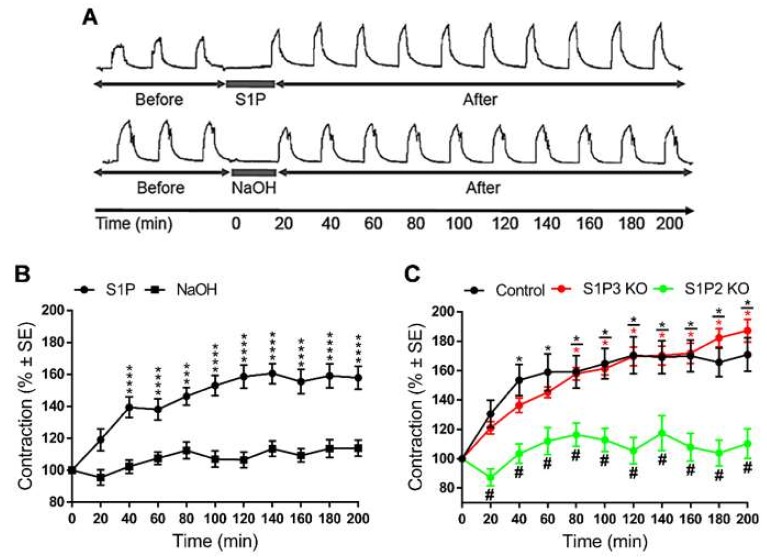
Long-term vascular effect of S1P. In TA segments prepared from WT mice repeated vasoconstriction responses to PE were enhanced after administration of S1P, and they remained elevated for three hours after the treatment. This effect did not occur if segments were treated with the vehicle (**A**,**B**). *** *p* < 0.001, **** *p* < 0.0001 vs. 0 min; Two-way ANOVA with Tukey’s *post hoc* test; (*n* = 15–18). Our next aim was to identify the receptor mediating the sustained potentiating effect of S1P on PE-induced contractions. Lack of S1P2 but not that of S1P3 abolished the S1P-induced increase in PE-mediated vasoconstrictions (**C**). S1P and PE were applied at 5 μM and 0.1 μM, respectively. * *p* < 0.05 vs. 0 min; # *p* < 0.05 vs. Control; Two-way ANOVA with Tukey’s *post hoc* test; (*n* = 15–28).

In all the aforementioned experiments, S1P-induced changes of α_1_-adrenergic vasoconstrictions were evaluated after washing out S1P from the organ chambers. Thus, exposure to S1P but not its continued presence induced changes in vascular reactivity. Therefore, an intriguing question was addressed: What duration of S1P exposure elicited enhancement of vascular reactivity? Interestingly, enhanced PE-induced contractions were detected for three hours after exposure to S1P but not to its vehicle (Figure 7A,B). In addition, similar to our observations in short-term experiments, this long-lasting vascular hyperreactivity failed to develop in S1P2 KO but remained unaltered in S1P3 KO vessels (Figure 7C).

## 3. Discussion

Contradictory reports have been published in the literature on the effect S1P has on vascular tone. Several reports described S1P-induced vasoconstriction, whereas others indicated vasodilation [9,32,44,45]. We also observed the vasorelaxant effect of S1P in precontracted murine thoracic aortas, which disappeared in eNOS KO vessels, indicating that the effect is mediated by endothelium-derived NO. Other studies have also reported a vasodilator effect of S1P via NO formation. For example, S1P was shown to increase NO production in cultured HUVEC [24] and in bovine lung microvascular endothelial cells [28]. Dantas et al. reported pertussis toxin (PTX)-sensitive eNOS activation by S1P in rat mesenteric arterioles [29].

There are divergent data in the literature concerning the receptor subtype mediating S1P-induced eNOS-dependent vasodilation. S1P was found to activate eNOS and promote NO release in rodent aortic rings, which proved to be mediated by S1P3 in mice [31,46]. A possible contribution of S1P1 has also been proposed. However, the lack of highly selective antagonists makes these results somewhat ambiguous [47]. On the other hand, S1P elicited eNOS activation in COS-7 cells in an S1P1-dependent manner [30]. Furthermore, VEGF was shown to increase S1P1 expression in aortic endothelial cells hyperreactivity and pretreatment of isolated vessels with VEGF-enhanced S1P-dependent vasodilation [48]. In rat mesenteric arterioles, S1P-induced dilatation inhibited the PI3K inhibitor wortmannin, and eNOS phosphorylation at Ser 1179 appeared to mediate the effect [29]. On the other hand, S1P1 agonist SEW2871 failed to induce any relaxation in the basilar, femoral, or mesenteric arteries of rats [47].

Reports on the vasoconstrictor effect of S1P and its signal transduction are also controversial. For example, S1P at a relatively high (micromolar) concentration was found to have a mild constrictor effect on isolated porcine pulmonary artery rings, whereas no S1P effect was observed at the same concentration in the aortas of the same species [49]. It was also reported that S1P induces contraction in isolated rat mesenteric and intrarenal vessels [46] and in canine coronaries [38], whereas vasoconstriction did not appear in rat carotid and femoral arteries [50] or in rat aortas [33]. Considerably lower S1P concentrations were reported to have a vasoconstrictor effect in canine, rat, murine and leporine basilar and middle cerebral arteries [36,41] and in rat portal veins [51]. Thus, the vascular effect of S1P appears to depend on the experimental animal species and on the vascular region, organ and tissues within the given species, as well as the local concentration of S1P. A further complicating issue is the use of different vehicles of S1P in these studies. It has been shown recently that the carrier of S1P may significantly modulate its biological effect, partly by biased agonism [43,52,53,54,55]. In our study, similar effects of S1P were observed in the absence and presence of albumin indicating that S1P may effectively bind to vascular smooth muscle S1P2 receptors even without any carriers.

There are many divergent results concerning the concentration of S1P in the circulatory system. The range of 100 nM–10 μM has been proposed depending on the species studied and the analytical method applied [56]. S1P is present at a concentration of approximately 100 nM in human lymph, whereas it has been reported to be as high as 1 μM in plasma [57]. Cells responsible for the systemic production of S1P are mainly the erythrocytes [50,58]. However, it has been reported that in the course of platelet activation, substantial amounts of S1P are released, leading to an elevation in its local concentration [59,60,61]. Thus, the S1P concentration of 5 µM applied in our experiments can rise locally, due mainly to platelet activation. Therefore, the vascular effects described in our present study are likely to develop in vascular disorders associated with platelet activation as has been shown during acute myocardial infarct and stroke.

The main finding of the present study is the demonstration of S1P-induced vascular hyperreactivity to α_1_-adrenergic stimulation of vascular smooth muscle cells. Our experiments revealed that the signaling pathway involves S1P2 receptor activation, followed by activation of G_12/13_ proteins, and consequently that of ROCK. This conclusion is supported by our observations that the potentiating effect of S1P is absent in the absence of S1P2 receptors or G_12/13_ proteins and can be abolished by the ROCK inhibitor Y-27632 and fasudil. Therefore, it is most likely that S1P treatment via activation of the ROCK pathway inhibits myosin phosphatase leading to a maintained state of myosin phosphorylation [62,63].

**Figure 8:**
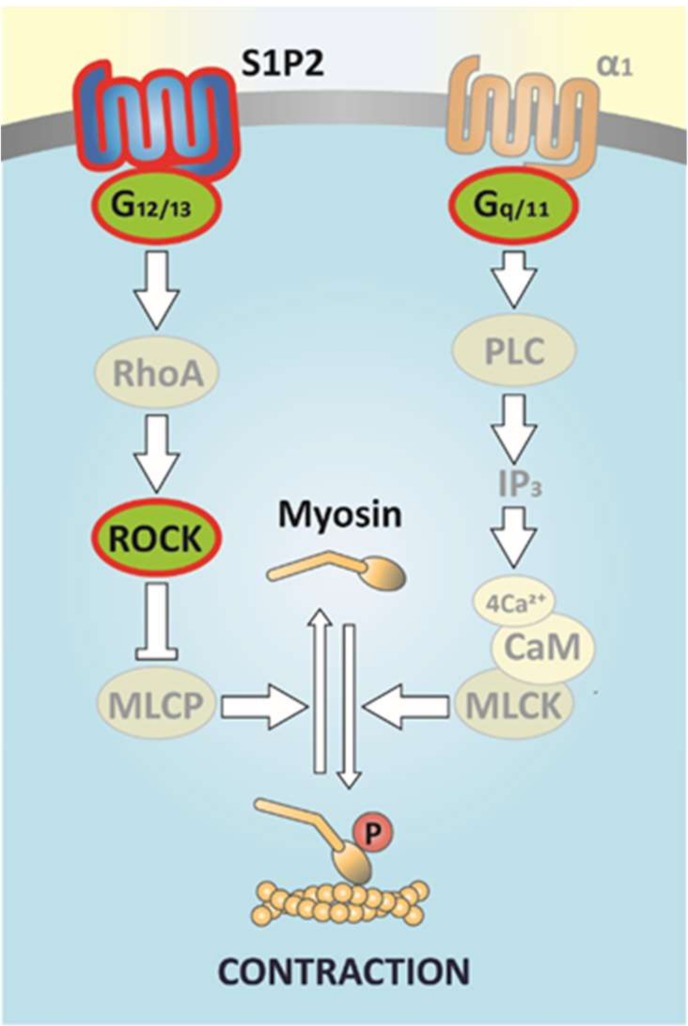
Scheme of the signaling pathway of S1P-elicted potentiation of α_1_-adrenergic vasoconstriction. Vasoconstriction induced by α_1_-adrenoreceptor activation is mediated exclusively by G_q/11_ signaling, which provokes intracellular Ca^2+^-release through PLC-derived IP_3_ production and consequent activation of myosin light chain kinase (MLCK) leading to activation of the cross-bridge cycle [61]. Meanwhile, S1P activates S1P2 receptors and the associated signaling pathway, involving G_12/13_ proteins, RhoA, and ROCK. In turn, ROCK inhibits myosin light chain phosphatase (MLCP), resulting in the retention of the phosphorylated state of myosin, which maintains the active cross-bridge cycle and allows for the development of sustained vasoconstriction. Signaling molecules identified in our previous [68] and present studies are highlighted.

This conclusion is consistent with previous findings indicating the role of ROCK in S1P-induced contraction in the pulmonary, uterine and skeletal muscle vasculature [64,65,66]. The intracellular pathways activated by S1P and α_1_-adrenergic receptors finally meet in the cross-bridge cycle where α_1_-adrenergic signaling drives myosin phosphorylation, whereas S1P2 signaling induces retention of the phosphorylated state of myosin, maintaining the active cross-bridge cycle and allowing for the development of sustained vasoconstriction (Figure 8). This interaction between α_1_-adrenergic and S1P2 signaling may be most relevant in the case of increased sympathetic activity in vascular regions expressing α_1_-adrenoreceptors. This conclusion is supported by the observation that PE-induced hypertension is significantly attenuated in S1P2 KO mice. In addition, mesenteric and renal blood flow increased significantly, whereas PE-induced elevation of vascular resistance was attenuated in anesthetized S1P2 KO mice, indicating decreased contractility in these vascular regions to α-adrenergic stimulation in vivo [67].

Finally, in order to study the duration of S1P-induced vascular hyperreactivity, identical doses of PE were applied at 20-minute intervals, and the magnitude of contraction responses was measured in WT, S1P2 KO or S1P3 KO vessels. On the one hand, our results confirmed that the effect of S1P is mediated by S1P2; on the other hand, the hyperreactivity was shown to be persistent, since the increase in contraction responses to PE remained detectable even three hours post-treatment in WT and S1P3 KO vessels. Taking into account that (i) large amounts of S1P may be released in the course of localized platelet activation, and (ii) the S1P effect identified in our work proved to be persistent, we propose that the mechanism described above may contribute to sustained vasoconstriction or even vasospasm in conditions associated with localized platelet activation and increased sympathetic tone such as what occurs during myocardial infarction and stroke. Therefore, the S1P2–G_12/13_–ROCK pathway appears to be a promising therapeutic target in thromboembolic disorders of the cardiovascular system [42].

## 4. Materials and Methods

All procedures were carried out according to the guidelines of the Hungarian Law of Animal Protection (28/1998) and were approved by the National Scientific Ethical Committee on Animal Experimentation (PEI/001/2706-13/2014, approval date: 17 December 2014).

### 4.1. Animals

C57BL/6 (WT) and eNOS knockout (eNOS KO) mice originated from Charles River Laboratories (Isaszeg, Hungary). S1P2 and S1P3 receptor-deficient animals (S1P2 KO; S1P3 KO) were generated and provided by Richard L. Proia (NIDDK, NIH, MD, USA). The G_12/13_ protein knockout (G_12/13_ KO) mouse line was described earlier [68]. Each mouse line had a C57BL/6 genetic background. As the eNOS KO and S1P3 KO strains have been back-crossed more than six times, age-matched WT animals of the C57BL/6 line served as controls. In the case of the S1P2 KO and G_12/13_ KO strains, animals originating from the same lines were tested as controls, with S1P^+/+^ and Gα_12_^+/+^/Gα_13_^fl/fl^ genotypes, respectively. Animals were housed in a temperature and light controlled room (12 h light-dark cycle, lights on at 7:00 a.m.), with free access to food and water.

### 4.2. Preparation of Vessels

For isolation of thoracic aortas, adult male mice were perfused transcardially with 10 mL heparinized (10 IU/mL) Krebs solution under deep ether anesthesia as previously described [69]. After removal, the aorta was cleaned of fat and loose connective tissue under a dissection microscope (M3Z; Wild Heerbrugg AG, Gais, Switzerland). Approximately 3-mm-long vascular segments were prepared and mounted on two parallel, horizontal stainless steel vessel wires (200 μm in diameter) of a myograph (610 M Multi Wire Myograph System; Danish Myo Technology A/S, Aarhus, Denmark). During preparation and mounting, special care was taken to preserve the integrity of the endothelium. Vessel rings were immersed in Krebs solution of the following composition (in mM): 119 NaCl, 4.7 KCl, 1.2 KH_2_PO_4_, 2.5 CaCl_2_·2H2O, 1.2 MgSO_4_·7H2O, 20 NaHCO_3_, 0.03 EDTA, and 10 glucose. Tissue baths were continuously bubbled with carbogen (95% O_2_ and 5% CO_2_), the temperature was set at 37 °C, and the pH was 7.4.

### 4.3. General Myography Protocol

During the experiments, the chambers of the myographs were filled with 6 ml Krebs solution and continuously aerated with carbogen gas. Every experiment started with a 30-minute resting period while the bath temperature reached 37 °C and the resting tone of the vessels was stabilized at 15 mN, which was determined to be optimal in a previous study [69]. We used different types of protocols for investigating the vasorelaxant and vasoconstrictor effects of S1P in this study. Nevertheless, in order to examine the reactivity and viability of the blood vessels, the same steps were taken at the beginning of each experiment. After the resting period, vessels were exposed to 124 mM K^+^ Krebs solution for one minute. This was followed by a series of washes with standard Krebs solution to reach the resting tone (15 mN). Then, the reactivity of the vessels was tested by administration of 10 μM PE and 0.1 μM acetylcholine (ACh). After multiple washings, when the tone of the vessels returned to the resting value, segments were exposed to 124 mM K^+^ Krebs solution for three minutes to elicit a maximal reference contraction. Thereafter, when the vessels had returned to resting tone, increasing concentrations of PE (0.1 nM to 10 μM) and Ach (1 nM to 10 μM) were given, in order to check smooth muscle activity and the integrity of the endothelium. This was also followed by a washing period.

### 4.4. Protocol for Testing the Direct Vasoactive Effect of S1P on Resting Tone and after PE Precontraction

The following protocol was used to examine the direct effect of S1P on the resting and the PE-induced vascular tone. Following the steps described above, S1P or NaOH was administered either on the resting tone or after precontraction by PE. Substances were left in the baths for 20 min to produce their effect, followed by repeated washing. At the end of the experiment, the vitality of the vessels was retested by administration of 124 mM K^+^.

### 4.5. Protocol for Testing the Short-Term Effect of S1P on α_1_-Adrenergic Vasoconstriction

In order to investigate the short-term effect of S1P on α_1_-adrenergic vasoconstriction, two PE dose-response curves were taken. After the first PE administration, the resting value of the vascular tone was restored by repeated washings. Thereafter, the vessels were exposed to S1P (5 µM) or its vehicle for 20 min. In most of the experiments 0.3 N NaOH was the vehicle of S1P. However, in a subset of experiments, 5% albumin was used in order to determine its potential influence on the effects of S1P. In part of the experiments, Y-27632 (2 µM) or fasudil (10 µM) was simultaneously applied with S1P. In a subset of the experiments, the thromboxane A_2_ analog U46619 (1 nM) was applied instead of two S1P PE dose-response curves, to examine if vasoconstriction can be enhanced by an S1P receptor-independent, the Rho kinase-dependent mechanism.

### 4.6. Protocol for Testing the Long-Term Effect of S1P on α_1_-Adrenergic Vasoconstriction

In our experiments, designed to explore the durability of the potentiating effect of S1P, the protocol was modified as follows. Following the initial evaluation of the reactivity of the vascular segments as described above, vasoconstrictive responses were provoked by repeated administration of PE every 20 min. After the response reached a stable level during three consecutive administrations, the vessels were exposed to S1P (5 µM) or its vehicle NaOH (0.3 N) for 20 min. Thereafter, the administration of PE was repeated every 20 minutes for three hours. At the end of the experiment, the vitality of the vessels was tested by administering 124 mM K^+^.

### 4.7. Reagents

Sphingosine-1-phosphate, U46619, and Y-27632 were purchased from Cayman Chemical Company (Ann Arbor, MI, USA). Fasudil hydrochloride, albumin, and all other drugs and chemicals used in the present study were purchased from Sigma-Aldrich (St. Louis, MO, USA). Stock solutions were made by dissolving the chemicals in 0.3 N sodium hydroxide (S1P), in 5% charcoal treated albumin (S1P), DMSO (U46619), distilled water (Y-27632, fasudil), or isotonic salt solution (PE, ACh).

### 4.8. Data Analysis

The MP100 System and AcqKnowledge 3.72 software from Biopac System, Inc. (Goleta, CA, USA) were used to record and analyze changes in the vascular tone. All data are presented as mean ± SE; “*n*” indicates the number of vessels tested in myograph experiments. For the statistical analysis, GraphPad Prism software (v.6.07; GraphPad Software Inc., La Jolla, CA, USA) was used. Dose-response curves for PE were plotted with responses expressed as percentage of the reference contraction induced by 124 mM K^+^ Krebs solution. Nonlinear regression was applied to compare the dose-response curves and determine E*max* and EC50 values. Student’s unpaired t-test was used when comparing the two variables. Unless otherwise indicated, all other comparisons between the different experimental groups were performed using ANOVA, followed by Tukey’s or Bonferroni’s *post hoc* tests. *p* < 0.05 was considered to be statistically significant.

## Supplementary Materials

Supplementary materials can be found at https://www.mdpi.com/1422-0067/20/24/6361/s1.

## Abbreviations

Ach	Acetylcholine
CTRL	Control
eNOS	Endothelial nitric oxide synthase
HUVEC	Human umbilical vein endothelial cell
IP3	Inositol trisphosphate
KO	Knockout
MLCP	Myosin light-chain phosphatase
MLCK	Myosin light-chain kinase
NO	Nitric oxide
PC	Precontraction
PE	Phenylephrine
PLC	Phospholipase C
PTX	Pertussis toxin
RT	Resting tone
ROCK	Rho-associated protein kinase
S1P	Sphingosine-1-phosphate
S1P1/S1P2/S1P3	Sphingosine-1-phosphate receptor
TA	Thoracic Aorta
W	Wash out
WT	Wild type

## References

[1] Kono M., Allende M.L., Proia R.L. (2008). Sphingosine-1-phosphate regulation of mammalian development. Biochim. Biophys. Acta.

[2] Mizugishi K., Li C., Olivera A., Bielawski J., Bielawska A., Deng C.X., Proia R.L. (2007). Maternal disturbance in activated sphingolipid metabolism causes pregnancy loss in mice. J. Clin. Investig..

[3] Maceyka M., Spiegel S. (2014). Sphingolipid metabolites in inflammatory disease. Nature.

[4] Spiegel S., Milstien S. (2011). The outs and the ins of sphingosine-1-phosphate in immunity. Nat. Rev. Immunol..

[5] Wollny T., Watek M., Durnas B., Niemirowicz K., Piktel E., Zendzian-Piotrowska M., Gozdz S., Bucki R. (2017). Sphingosine-1-Phosphate Metabolism and Its Role in the Development of Inflammatory Bowel Disease. Int. J. Mol. Sci..

[6] Cannavo A., Liccardo D., Komici K., Corbi G., de Lucia C., Femminella G.D., Elia A., Bencivenga L., Ferrara N., Koch W.J. (2017). Sphingosine Kinases and Sphingosine 1-Phosphate Receptors: Signaling and Actions in the Cardiovascular System. Front. Pharmacol..

[7] Levkau B. (2013). Cardiovascular effects of sphingosine-1-phosphate (S1P). Handb. Exp. Pharmacol..

[8] Arkensteijn B.W., Berbee J.F., Rensen P.C., Nielsen L.B., Christoffersen C. (2013). The apolipoprotein m-sphingosine-1-phosphate axis: Biological relevance in lipoprotein metabolism, lipid disorders and atherosclerosis. Int. J. Mol. Sci..

[9] Kerage D., Brindley D.N., Hemmings D.G. (2014). Review: Novel insights into the regulation of vascular tone by sphingosine 1-phosphate. Placenta.

[10] Hemmings D.G. (2006). Signal transduction underlying the vascular effects of sphingosine 1-phosphate and sphingosylphosphorylcholine. Naunyn Schmiedeberg Arch. Pharmacol..

[11] Pelletier D., Hafler D.A. (2012). Fingolimod for multiple sclerosis. N. Engl. J. Med..

[12] Brinkmann V., Billich A., Baumruker T., Heining P., Schmouder R., Francis G., Aradhye S., Burtin P. (2010). Fingolimod (FTY720): Discovery and development of an oral drug to treat multiple sclerosis. Nat. Rev. Drug Discov..

[13] Pujol-Lereis L.M. (2019). Alteration of Sphingolipids in Biofluids: Implications for Neurodegenerative Diseases. Int. J. Mol. Sci..

[14] Patmanathan S.N., Wang W., Yap L.F., Herr D.R., Paterson I.C. (2017). Mechanisms of sphingosine 1-phosphate receptor signalling in cancer. Cell. Signal..

[15] Pyne N.J., Tonelli F., Lim K.G., Long J.S., Edwards J., Pyne S. (2012). Sphingosine 1-phosphate signalling in cancer. Biochem. Soc. Trans..

[16] Pyne N.J., El Buri A., Adams D.R., Pyne S. (2018). Sphingosine 1-phosphate and cancer. Adv. Biol. Regul..

[17] Mahajan-Thakur S., Bien-Moller S., Marx S., Schroeder H., Rauch B.H. (2017). Sphingosine 1-phosphate (S1P) signaling in glioblastoma multiforme-A systematic review. Int. J. Mol. Sci..

[18] Zhuang X.P., Zhu Q. (2016). Sphingosine-1-phosphate/sphingosine-1-phosphate receptor 1 and T cell migration. Acta Pharm. Sin..

[19] Tiper I.V., East J.E., Subrahmanyam P.B., Webb T.J. (2016). Sphingosine 1-phosphate signaling impacts lymphocyte migration, inflammation and infection. Pathog. Dis..

[20] Paik J.H., Chae S., Lee M.J., Thangada S., Hla T. (2001). Sphingosine 1-phosphate-induced endothelial cell migration requires the expression of EDG-1 and EDG-3 receptors and Rho-dependent activation of alpha vbeta3- and beta1-containing integrins. J. Biol. Chem..

[21] Lemos J.P., Smaniotto S., Messias C.V., Moreira O.C., Cotta-de-Almeida V., Dardenne M., Savino W., Mendes-da-Cruz D.A. (2018). Sphingosine-1-Phosphate Receptor 1 Is Involved in Non-Obese Diabetic Mouse Thymocyte Migration Disorders. Int. J. Mol. Sci..

[22] Takuwa Y., Du W., Qi X., Okamoto Y., Takuwa N., Yoshioka K. (2010). Roles of sphingosine-1-phosphate signaling in angiogenesis. World J. Biol. Chem..

[23] Kono M., Mi Y., Liu Y., Sasaki T., Allende M.L., Wu Y.P., Yamashita T., Proia R.L. (2004). The sphingosine-1-phosphate receptors S1P1, S1P2, and S1P3 function coordinately during embryonic angiogenesis. J. Biol. Chem..

[24] Wilkerson B.A., Argraves K.M. (2014). The role of sphingosine-1-phosphate in endothelial barrier function. Biochim. Et Biophys. Acta.

[25] Xiong Y., Hla T. (2014). S1P control of endothelial integrity. Curr. Top. Microbiol. Immunol..

[26] Sanchez T., Skoura A., Wu M.T., Casserly B., Harrington E.O., Hla T. (2007). Induction of vascular permeability by the sphingosine-1-phosphate receptor-2 (S1P2R) and its downstream effectors ROCK and PTEN. Arterioscler. Thromb. Vasc. Biol..

[27] Kimura T., Sato K., Kuwabara A., Tomura H., Ishiwara M., Kobayashi I., Ui M., Okajima F. (2001). Sphingosine 1-phosphate may be a major component of plasma lipoproteins responsible for the cytoprotective actions in human umbilical vein endothelial cells. J. Biol. Chem..

[28] Morales-Ruiz M., Lee M.J., Zollner S., Gratton J.P., Scotland R., Shiojima I., Walsh K., Hla T., Sessa W.C. (2001). Sphingosine 1-phosphate activates Akt, nitric oxide production, and chemotaxis through a Gi protein/phosphoinositide 3-kinase pathway in endothelial cells. J. Biol. Chem..

[29] Dantas A.P., Igarashi J., Michel T. (2003). Sphingosine 1-phosphate and control of vascular tone. Am. J. Physiol. Heart Circ. Physiol..

[30] Igarashi J., Michel T. (2000). Agonist-modulated targeting of the EDG-1 receptor to plasmalemmal caveolae. eNOS activation by sphingosine 1-phosphate and the role of caveolin-1 in sphingolipid signal transduction. J. Biol. Chem..

[31] Nofer J.R., van der Giet M., Tolle M., Wolinska I., von Wnuck Lipinski K., Baba H.A., Tietge U.J., Godecke A., Ishii I., Kleuser B. (2004). HDL induces NO-dependent vasorelaxation via the lysophospholipid receptor S1P3. J. Clin. Investig..

[32] Igarashi J., Michel T. (2009). Sphingosine-1-phosphate and modulation of vascular tone. Cardiovasc. Res..

[33] Coussin F., Scott R.H., Wise A., Nixon G.F. (2002). Comparison of sphingosine 1-phosphate-induced intracellular signaling pathways in vascular smooth muscles: Differential role in vasoconstriction. Circ. Res..

[34] Bischoff A., Czyborra P., Fetscher C., Meyer Zu Heringdorf D., Jakobs K.H., Michel M.C. (2000). Sphingosine-1-phosphate and sphingosylphosphorylcholine constrict renal and mesenteric microvessels in vitro. Br. J. Pharmacol..

[35] Ohmori T., Yatomi Y., Osada M., Kazama F., Takafuta T., Ikeda H., Ozaki Y. (2003). Sphingosine 1-phosphate induces contraction of coronary artery smooth muscle cells via S1P2. Cardiovasc. Res..

[36] Tosaka M., Okajima F., Hashiba Y., Saito N., Nagano T., Watanabe T., Kimura T., Sasaki T. (2001). Sphingosine 1-phosphate contracts canine basilar arteries in vitro and in vivo: Possible role in pathogenesis of cerebral vasospasm. Stroke.

[37] Bolz S.S., Vogel L., Sollinger D., Derwand R., de Wit C., Loirand G., Pohl U. (2003). Nitric oxide-induced decrease in calcium sensitivity of resistance arteries is attributable to activation of the myosin light chain phosphatase and antagonized by the RhoA/Rho kinase pathway. Circulation.

[38] Sugiyama A., Yatomi Y., Ozaki Y., Hashimoto K. (2000). Sphingosine 1-phosphate induces sinus tachycardia and coronary vasoconstriction in the canine heart. Cardiovasc. Res..

[39] Bischoff A., Czyborra P., Meyer Zu Heringdorf D., Jakobs K.H., Michel M.C. (2000). Sphingosine-1-phosphate reduces rat renal and mesenteric blood flow in vivo in a pertussis toxin-sensitive manner. Br. J. Pharmacol..

[40] Tolle M., Levkau B., Kleuser B., van der Giet M. (2007). Sphingosine-1-phosphate and FTY720 as anti-atherosclerotic lipid compounds. Eur. J. Clin. Investig..

[41] Salomone S., Yoshimura S., Reuter U., Foley M., Thomas S.S., Moskowitz M.A., Waeber C. (2003). S1P3 receptors mediate the potent constriction of cerebral arteries by sphingosine-1-phosphate. Eur. J. Pharmacol..

[42] Blaho V.A., Hla T. (2014). An update on the biology of sphingosine 1-phosphate receptors. J. Lipid Res..

[43] Takahashi C., Kurano M., Nishikawa M., Kano K., Dohi T., Miyauchi K., Daida H., Shimizu T., Aoki J., Yatomi Y. (2017). Vehicle-dependent Effects of Sphingosine 1-phosphate on Plasminogen Activator Inhibitor-1 Expression. J. Atheroscler. Thromb..

[44] Michel M.C., Mulders A.C., Jongsma M., Alewijnse A.E., Peters S.L. (2007). Vascular effects of sphingolipids. Acta Paediatr..

[45] Levkau B. (2008). Sphingosine-1-phosphate in the regulation of vascular tone: A finely tuned integration system of S1P sources, receptors, and vascular responsiveness. Circ. Res..

[46] Tolle M., Levkau B., Keul P., Brinkmann V., Giebing G., Schonfelder G., Schafers M., von Wnuck Lipinski K., Jankowski J., Jankowski V. (2005). Immunomodulator FTY720 Induces eNOS-dependent arterial vasodilatation via the lysophospholipid receptor S1P3. Circ. Res..

[47] Waeber C. (2013). Sphingosine 1-phosphate (S1P) signaling and the vasculature. Lysophospholipid Recept. Signal. Biochem..

[48] Igarashi J., Erwin P.A., Dantas A.P., Chen H., Michel T. (2003). VEGF induces S1P1 receptors in endothelial cells: Implications for cross-talk between sphingolipid and growth factor receptors. Proc. Natl. Acad. Sci. USA.

[49] Hsiao S.-H., Constable P.D., Smith G.W., Haschek W.M. (2005). Effects of Exogenous Sphinganine, Sphingosine, and Sphingosine-1-Phosphate on Relaxation and Contraction of Porcine Thoracic Aortic and Pulmonary Arterial Rings. Toxicol. Sci..

[50] Xiong Y., Yang P., Proia R.L., Hla T. (2014). Erythrocyte-derived sphingosine 1-phosphate is essential for vascular development. J. Clin. Investig..

[51] Ikeda H., Nagashima K., Yanase M., Tomiya T., Arai M., Inoue Y., Tejima K., Nishikawa T., Watanabe N., Omata M. (2004). Sphingosine 1-phosphate enhances portal pressure in isolated perfused liver via S1P2 with Rho activation. Biochem. Biophys. Res. Commun..

[52] Wilkerson B.A., Grass G.D., Wing S.B., Argraves W.S., Argraves K.M. (2012). Sphingosine 1-phosphate (S1P) carrier-dependent regulation of endothelial barrier: High density lipoprotein (HDL)-S1P prolongs endothelial barrier enhancement as compared with albumin-S1P via effects on levels, trafficking, and signaling of S1P1. J. Biol. Chem..

[53] Kurano M., Hara M., Tsuneyama K., Sakoda H., Shimizu T., Tsukamoto K., Ikeda H., Yatomi Y. (2014). Induction of insulin secretion by apolipoprotein M, a carrier for sphingosine 1-phosphate. Biochim. Biophys. Acta.

[54] Blaho V.A., Galvani S., Engelbrecht E., Liu C., Swendeman S.L., Kono M., Proia R.L., Steinman L., Han M.H., Hla T. (2015). HDL-bound sphingosine-1-phosphate restrains lymphopoiesis and neuroinflammation. Nature.

[55] Galvani S., Sanson M., Blaho V.A., Swendeman S.L., Obinata H., Conger H., Dahlback B., Kono M., Proia R.L., Smith J.D. (2015). HDL-bound sphingosine 1-phosphate acts as a biased agonist for the endothelial cell receptor S1P1 to limit vascular inflammation. Sci. Signal..

[56] Hanel P., Andreani P., Graler M.H. (2007). Erythrocytes store and release sphingosine 1-phosphate in blood. FASEB J..

[57] Proia R.L., Hla T. (2015). Emerging biology of sphingosine-1-phosphate: Its role in pathogenesis and therapy. J. Clin. Investig..

[58] Pappu R., Schwab S.R., Cornelissen I., Pereira J.P., Regard J.B., Xu Y., Camerer E., Zheng Y.W., Huang Y., Cyster J.G. (2007). Promotion of lymphocyte egress into blood and lymph by distinct sources of sphingosine-1-phosphate. Science.

[59] Camerer E., Regard J.B., Cornelissen I., Srinivasan Y., Duong D.N., Palmer D., Pham T.H., Wong J.S., Pappu R., Coughlin S.R. (2009). Sphingosine-1-phosphate in the plasma compartment regulates basal and inflammation-induced vascular leak in mice. J. Clin. Investig..

[60] Yatomi Y., Ozaki Y., Ohmori T., Igarashi Y. (2001). Sphingosine 1-phosphate: Synthesis and release. Prostaglandins Other Lipid Mediat..

[61] Venkataraman K., Lee Y.M., Michaud J., Thangada S., Ai Y., Bonkovsky H.L., Parikh N.S., Habrukowich C., Hla T. (2008). Vascular endothelium as a contributor of plasma sphingosine 1-phosphate. Circ. Res..

[62] Szasz T., Webb R.C. (2017). Rho-Mancing to Sensitize Calcium Signaling for Contraction in the Vasculature: Role of Rho Kinase. Adv. Pharmacol..

[63] Liu Z., Khalil R.A. (2018). Evolving mechanisms of vascular smooth muscle contraction highlight key targets in vascular disease. Biochem. Pharmacol..

[64] Bolz S.S., Vogel L., Sollinger D., Derwand R., Boer C., Pitson S.M., Spiegel S., Pohl U. (2003). Sphingosine kinase modulates microvascular tone and myogenic responses through activation of RhoA/Rho kinase. Circulation.

[65] Hemmings D.G., Hudson N.K., Halliday D., O’Hara M., Baker P.N., Davidge S.T., Taggart M.J. (2006). Sphingosine-1-phosphate acts via rho-associated kinase and nitric oxide to regulate human placental vascular tone. Biol. Reprod..

[66] Szczepaniak W.S., Pitt B.R., McVerry B.J. (2010). S1P2 receptor-dependent Rho-kinase activation mediates vasoconstriction in the murine pulmonary circulation induced by sphingosine 1-phosphate. Am. J. Physiol. Lung Cell. Mol. Physiol..

[67] Lorenz J.N., Arend L.J., Robitz R., Paul R.J., MacLennan A.J. (2007). Vascular dysfunction in S1P2 sphingosine 1-phosphate receptor knockout mice. Am. J. Physiol. Regul. Integr. Comp. Physiol..

[68] Wirth A., Benyo Z., Lukasova M., Leutgeb B., Wettschureck N., Gorbey S., Orsy P., Horvath B., Maser-Gluth C., Greiner E. (2008). G12-G13-LARG-mediated signaling in vascular smooth muscle is required for salt-induced hypertension. Nat. Med..

[69] Horvath B., Orsy P., Benyo Z. (2005). Endothelial NOS-mediated relaxations of isolated thoracic aorta of the C57BL/6J mouse: A methodological study. J. Cardiovasc. Pharmacol..

