# Supplementary material for: Sphingosine-1-Phosphate Enhances α1-Adrenergic Vasoconstriction via S1P2–G12/13–ROCK Mediated Signaling
Source: Int J Mol Sci. 2019 Dec 17;20(24):6361. doi: 10.3390/ijms20246361 (PMC6941080; doi:10.3390/ijms20246361)
Supplement: Supplementary file 1 [file ijms-20-06361-s001.pdf]

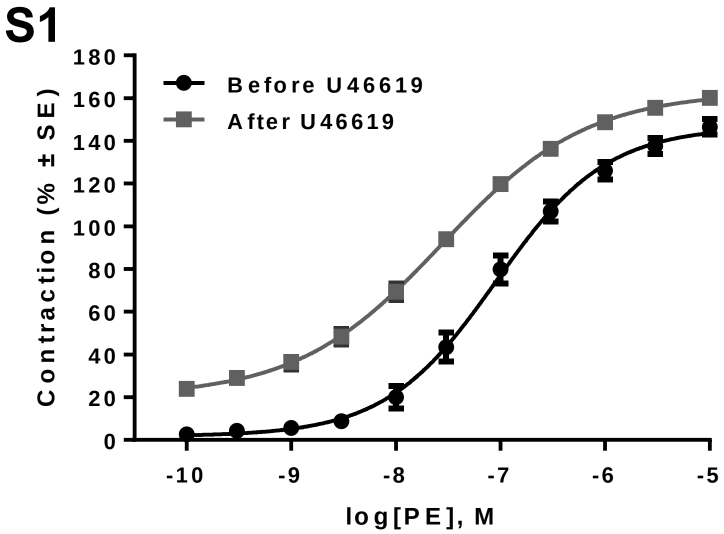

	Before U46619	After U46619
E_{max}	147.9±5.3	163.2±3.6 *
$LogEC_{50}$	-7.04±0.07	-7.56±0.07 *

Figure S1. Effects of the thromboxane prostanoid (TP) receptor agonist U46619 on α_1-adrenoreceptor-mediated vasoconstriction in S1P2 KO vessels. U46619 (1 nM) was able to further enhance the contraction responses in spite of the relatively high baseline reactivity of the S1P2 KO vessels to phenylephrine (PE). *P < 0.05 vs. before U46619 (n=15).

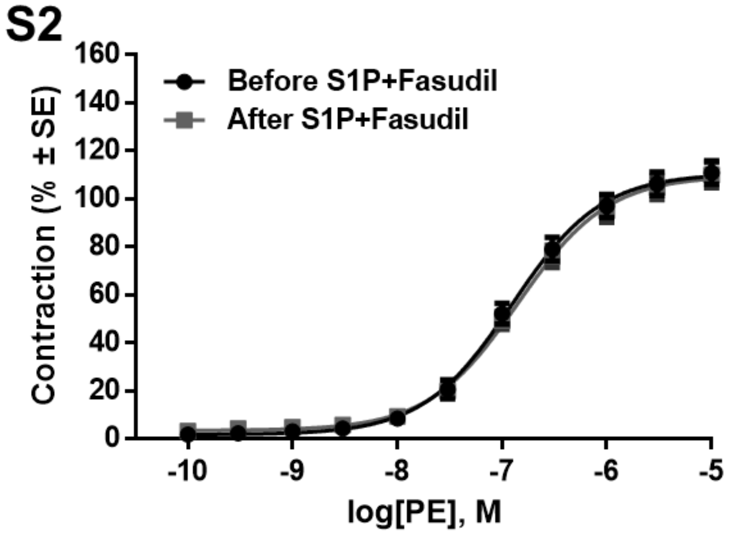

	Before S1P+Fasudil	After S1P+Fasudil
E_{max}	110.7±3.0	109.8±2.9
$LogEC_{50}$	-6.90±0.05	-6.84±0.04

Figure S2. The effect of fasudil on S1P-induced potentiation of α_1-adrenoreceptor-mediated vasoconstriction. Co-administration of the Rho kinase inhibitor fasudil (10 μM) was also able to prevent the S1P-induced potentiating effect similar to that of Y-27632. *P < 0.05 vs. before S1P (n=11).
